# Healing and Angiogenic Properties of Collagen/Chitosan Scaffolds Enriched with Hyperstable FGF2-STAB^®^ Protein: In Vitro, Ex Ovo and In Vivo Comprehensive Evaluation

**DOI:** 10.3390/biomedicines9060590

**Published:** 2021-05-22

**Authors:** Lucy Vojtová, Veronika Pavliňáková, Johana Muchová, Katarína Kacvinská, Jana Brtníková, Martin Knoz, Břetislav Lipový, Martin Faldyna, Eduard Göpfert, Jakub Holoubek, Zdeněk Pavlovský, Monika Vícenová, Veronika Hefka Blahnová, Vanessa Hearnden, Eva Filová

**Affiliations:** 1 CEITEC–Central European Institute of Technology, Brno University of Technology, 612 00 Brno, Czech Republic; lucy.vojtova@ceitec.vutbr.cz (L.V.); johana.muchova@ceitec.vutbr.cz (J.M.); katarina.kacvinska@ceitec.vutbr.cz (K.K.); Jana.Brtnikova@ceitec.vutbr.cz (J.B.); bretalipovy@gmail.com (B.L.); 2 Faculty of Medicine, Department of Burns and Plastic Surgery, Institution Shared with the University Hospital Brno, 625 00 Brno, Czech Republic; martinknoz@gmail.com (M.K.); holoubekjakub@yahoo.com (J.H.); 3 Clinic of Plastic and Esthetic Surgery, St Anne’s University Hospital, 602 00 Brno, Czech Republic; 4 Veterinary Research Institute, 621 00 Brno, Czech Republic; faldyna@vri.cz (M.F.); gopfert@vri.cz (E.G.); vicenova@vri.cz (M.V.); 5 Faculty of Medicine, Institute of Pathology, University Hospital Brno, Masaryk University, 625 00 Brno, Czech Republic; pavlovsky.zdenek@fnbrno.cz; 6 Institute of Experimental Medicine of the Czech Academy of Science, 142 20 Prague, Czech Republic; veronika.blahnova@iem.cas.cz (V.H.B.); eva.filova@iem.cas.cz (E.F.); 7 Department of Materials Science and Engineering, Kroto Research Institute, North Campus, University of Sheffield, Broad Lane, Sheffield S3 7HQ, UK; v.hearnden@sheffield.ac.uk

**Keywords:** collagen, chitosan, scaffold, FGF2, skin regeneration, tissue engineering

## Abstract

Wound healing is a process regulated by a complex interaction of multiple growth factors including fibroblast growth factor 2 (FGF2). Although FGF2 appears in several tissue engineered studies, its applications are limited due to its low stability both in vitro and in vivo. Here, this shortcoming is overcome by a unique nine-point mutant of the low molecular weight isoform FGF2 retaining full biological activity even after twenty days at 37 °C. Crosslinked freeze-dried 3D porous collagen/chitosan scaffolds enriched with this hyper stable recombinant human protein named FGF2-STAB^®^ were tested for in vitro biocompatibility and cytotoxicity using murine 3T3-A31 fibroblasts, for angiogenic potential using an ex ovo chick chorioallantoic membrane assay and for wound healing in vivo with 3-month old white New Zealand rabbits. Metabolic activity assays indicated the positive effect of FGF2-STAB^®^ already at very low concentrations (0.01 µg/mL). The angiogenic properties examined ex ovo showed enhanced vascularization of the tested scaffolds. Histological evaluation and gene expression analysis by RT-qPCR proved newly formed granulation tissue at the place of a previous skin defect without significant inflammation infiltration in vivo. This work highlights the safety and biocompatibility of newly developed crosslinked collagen/chitosan scaffolds involving FGF2-STAB^®^ protein. Moreover, these sponges could be used as scaffolds for growing cells for dermis replacement, where neovascularization is a crucial parameter for successful skin regeneration.

## 1. Introduction

The skin is the largest organ of the body and represents the outer covering of the human body. Its main function is to provide a barrier between the internal environment of the body and the external agents. Naturally, the skin is exposed to various injuries including mechanical damage, microbiological attacks, and physical and chemical effects, e.g., burns [1]. Therefore, for the survival of the organism, it is necessary to ensure a suitable regeneration of the skin, whether by natural healing processes or by means of artificial medical interventions. Skin is a complex tissue consisting of the epidermis, dermis, subcutaneous tissue (hypodermis), and skin appendages (hair follicles, nails, sweat glands and sebaceous glands) [2]. The keratinocytes are the major cell component of the external layer, the epidermis, forming a keratinized stratified squamous epithelium [3]. The more heterogeneous layer, the dermis, consists of an extracellular matrix (ECM) containing mainly collagen (Coll) produced by fibroblasts. Skin wound healing is a complex process including an interaction between cells, mediators, cytokines, matrix remodeling, and the neurovascular system. The success rate of skin healing has significantly increased with the use of tailored skin substitutes [4]. The main idea of scaffolds is to imitate the skin ECM and its properties (e.g., strength, elasticity, and flexibility) [5]. Scaffolds must meet some requirements, such as biocompatibility, biodegradability, temporary mechanical support, permeability, and suitable interconnecting pores appropriate to support cell integration [6,7]. Biomaterials such as Coll [8] and its hydrolyzed form—gelatin [9], various proteoglycans [10], hyaluronic acid [11], alginate-based substrates [12] and chitosan (Chit) [13] are widely used in the scaffold production for tissue engineering. These natural polymers have many excellent properties; they are biodegradable, biologically active and promote outstanding cell adhesion and growth [14]. On the other hand, the biobased scaffolds generally have poor mechanical properties, which limits their potential applications in the biomedicine [15].

Coll is the most abundant protein in the human body where it acts as a structural building block of the ECM found in most native tissues. Coll exhibits some unique structural properties important for skin tissue engineering applications: they transmit forces, dissipate energy, prevent premature mechanical failure, and provide biological signals to adjacent cells that regulate functional responses [16,17]. Coll is also low inflammatory, biodegradable, biocompatible, and bioresorbable biopolymer [18]. It has high water affinity, good cell compatibility, low antigenicity, and ability to promote tissue regeneration [19]. However, pure Coll scaffolds lack structural stability upon hydration and mechanical strength, which limit their applications in particular tissues [20]. Improvement of the mechanical properties of Coll scaffolds can be achieved by intermolecular crosslinking using physical or chemical methods [21]. Moreover, a mixing of Coll with other materials, such as Chit, is also often used to enhance the mechanical strength of Coll scaffolds [22].

Chit exhibits various interesting biological properties such as biocompatibility, good interaction with cells, lack of immunogenicity, and antimicrobial activity [23,24]. Moreover, Chit also enhances the wound healing process via improving the functions of macrophages, fibroblasts, and inflammatory cells [25]. It has been shown that Coll can be synergistically used with Chit to achieve better biological results [22,26,27]. Such a combination also promotes the proliferation [28] as well as angiogenesis [29]. Moreover, various organic additives, including, e.g., proteins or growth factor, can be used to improve properties of materials based on Coll/Chit mixtures [30,31].

Fibroblast growth factor 2 (FGF2), also known as basic fibroblast growth factor, is one of the family of mammalian fibroblast growth factors consisting of at least 23 members (22 identified in humans to date) [32,33]. FGF2 is a polypeptide mitogen stimulating cell viability associated with wound healing [34]. FGF2 also plays a role in the mitogenesis of mesenchymal cells [24]. FGF2 is a commonly used growth accelerator for tympanic membrane perforation [35]. FGF2 expression, gene function, and in vitro activity was revealed in brain, peripheral nerves, smooth muscle, skeletal muscle, heart, angiogenesis, hematopoietic cells, chondrocytes, and osteoblasts [36]. Momose et al. [37] confirmed the excellent biocompatibility of an FGF2 loaded Coll scaffold. A scaffold including FGF2 promoted rapid tissue growth in the inner spaces of the scaffold [38]. Yang et al. [39] demonstrated that the targeted delivery of FGF2 to subchondral bone significantly improved the repair of articular cartilage defects. FGF2 was also incorporated in gelatin hydrogel particles in a Coll sponge [40]. This system was used for the dentin defects in amputated dental pulp and found that the controlled release of FGF2 from gelatin particles induced the regeneration of dental pulp with a dentin layer. Fayazzadeh et al. [41] stated that FGF2 significantly decreased the percentage of flap necrosis and improved the percentage of the ischemic survival area. The use of FGF2 increased skin elasticity and hydration of the skin, decreased the depth of wrinkles and the depth of pigmentation and supported hair growth [42,43]. On the other hand, the applications of FGF2 are limited due to its low stability in vivo and in vitro [44].

In our previous work [23], we extensively analyzed 3D porous Coll-based scaffolds modified with polysaccharides (calcium salt of oxidized cellulose, Chit, and chitin/chitosan-glucan complex) and enriched with bovine platelet lysate, regarding their chemical, mechanical and biological properties. In this study, depending on the in vitro results, we applied stabilized FGF2 in Coll/Chit scaffolds for a full-thickness skin replacement on an animal model. A unique nine-point mutant of low molecular weight isoform FGF2 with in vitro functional half time at 37 °C more than 20 days was used. The engineering process of this FGF2-STAB^®^ is described in [45]. We hypothesized that a combination of FGF2-STAB^®^ and Coll/Chit-based scaffolds significantly improved the biological effects for the skin tissue engineering. Accordingly, we investigated the optimal FGF2-STAB^®^ concentration in the scaffold, which is crucial for the best in vivo results. The scaffolds were implanted into rabbit skin defects as a preclinical test to assess the safety and biological effects of scaffold with FGF2-STAB^®^.

## 2. Materials and Methods

### 2.1. Scaffold Preparation

Coll (type I, 8% aqueous solution, Collado s.r.o., Brno, Czech Republic) was freeze-dried to obtain 100% Coll foam. Chit from crab shells (highly viscous, 2-Amino-2-deoxy-(1→4)-β-D-glucopyranan, Poly-(1,4-β-D-glucopyranosamin), (Sigma-Aldrich, Darmstadt, Germany) was used as received without further purification. Coll/Chit scaffolds were prepared by freeze-drying method from 0.5 wt % Coll aqueous suspension (Milli-Q^®^, ultrapure water-type II according to ISO 3696 being prepared on Elix 5 UV Water Purification System, Merck, Darmstadt, Germany) and followed by crosslinking process with the mixture of *N*-(3-Dimethylaminopropyl)-*N*’-ethylcarbodiimide hydrochloride (EDC, Sigma-Aldrich) and *N*-hydroxysuccinimide (NHS, Sigma-Aldrich, Darmstadt, Germany) crosslinkers according to our previous work [46]. Briefly, the calculated amount of Chit was slowly added to a cold (4 °C) Coll aqueous suspension with a concentration of 0.5 wt %. Consequently, the mixtures were homogenized and frieze-dried in Martin Christ Epsilon 2-10D lyophilizator (Osterode am Harz, Germany) at 35 °C under 1 mBar for 15 h followed by a secondary drying process at 25 °C under 0.01 mBar until decreasing Δp (the change in pressure was up to 10%). Post-crosslinking process with carbodiimides system (EDC/NHS with molar ratio of 2/1) insisted 2 h followed by washing twice with 0.1 M Na_2_HPO_4_ and ultrapure water for removal byproducts. Finally, FGF2- STAB^®^ was implemented in the scaffolds. Stable FGF2-STAB^®^ (recombinant human protein, *E. coli*-derived, His-tagged; >95% purity) was obtained from Enantis, s.r.o. (Brno, Czech Republic). An adequate volume of cold (4 °C) FGF2-STAB^®^ (0, 0.01, 0.05, 0.1, 0.5, 1, 5, 10, 50 and 100 µg/mL) was vigorously stirred and left to soak into a scaffold for 10 min and consequently freeze-dried again. Table 1 sums up the composition of the prepared scaffolds as well as their labelling. Sample labeling of the prepared scaffolds were formed by the abbreviations of their composition with the used concentration of FGF2-STAB^®^ (e.g., Coll/Chit_0.01 FGF2).

### 2.2. Swelling Ratio

The material was cut into 1 × 2 cm strips and immersed into ultrapure water. Every piece of sample was weighted in its dry state before immersion. The weight was also recorded after gently removing the surface water with filter paper at several intervals up to 3 h. The swelling ratio (*SR*) was calculated according to Equation (1), where *W_s_* is the weight of the sample in swollen state and *W_i_* is the initial weight of dry sample.

*SR = W_s_/W_i_* (1)

### 2.3. Hydrolytic Degradation

After swelling measurement, the samples were placed into the vials containing ultrapure water. The hydrolytic degradation was simulated in an incubator at 37 °C. The samples were removed, surplus water from the surface was removed and the samples were weighed every day for one week and then after approximately 1 month. The degradation solution was changed for freshness after every measurement.

The degradation was calculated according to Equation (2), where the *W_D_* is the weight of swollen scaffold in the appropriate day and the *W* is the stabilized weight at 60 min of swelling at first day of the measurement.

[%] = 100 − (*W_D_* × 100/*W*) (2)

### 2.4. Enzymatic Degradation

The enzymatic degradation was examined in vitro with a solution of collagenase from *Clostridium histolyticum* (≥125 CDU/mg, lyophilized powder, Sigma-Aldrich, Darmstadt, Germany) or lysozyme (human, recombinant, expressed in rice—120,530 units/mg protein, lyophilized powder, Sigma-Aldrich, Darmstadt, Germany) in phosphate buffered saline (PBS) at physiological pH 7.4 at 37 °C. Corresponding amount of enzyme was dissolved in PBS to obtain a solution with collagenase concentration of 2.2 mg·dm^−3^. The solution was then kept at 37 °C for 1 h. Scaffold samples were immersed in PBS for 10 min (according to the swelling behavior results) at 37 °C, deprived of excess water, and weighed in a wet state. Afterwards, the PBS solution with collagenase was poured over the sample. For degradation experiment, samples were put into an incubator (37 °C) to simulate the physiological conditions. Each sample was removed from the solution, dried with filtration paper, and weighed in 2, 4, 8, 24, 48, 72 and 144 h intervals from the first immersion. To calculate the weight loss of each sample, the obtained data were put into Equation (3), where *W_L_* indicates the mass loss, *w_i_* the initial weight of a wet scaffold after PBS swelling, and *w_t_* mass of that scaffold after the corresponding elapsed time.

*W_L_* [%] = (*w_i_* − *w_t_*)/*w_i_* × 100 (3)

### 2.5. Scaffold Morphology

Scaffold morphology was investigated employing a scanning electron microscope (SEM, Tescan MIRA3, Brno, Czech Republic). All observations were made in the secondary electron emission mode with a high voltage of 10 kV. For better resolution, the scaffolds were coated with the 20 nm gold layer. Pore size of the prepared scaffolds was calculated from the SEM images using ImageJ software. Pores were evaluated from 5 different SEM images of each scaffold with a view field of 1.5 mm. Average pore size was calculated using 150 measured values from SEM images.

### 2.6. Mechanical Testing

Samples prepared in 12 × 12 cm plastic plates were cut into strips of 4 cm length, 10 mm width (approximately 3 mm thickness) and were liable to Dynamical mechanical analysis (DMA) with a model instrument Rheometer RSAG2 (TA Instruments Inc., New Castle, DE, USA). The samples were separately mounted into the grip in a central position. First biomechanical testing was carried out at room temperature (23 °C), while the second testing involved constant hydrating conditions, where the samples were immersed in PBS at 37 °C in a built chamber, surrounding the grips. Before every measurement, 10 min of swelling was provided for every sample. Because the stresses in both directions perpendicular to the axial of the specimen are zero, the elastic modulus E was determined by the ratio of stress to strain using Electro Force DMA Data Analysis program (TA Instruments Inc., New Castle, DE, USA).

### 2.7. Cells and Culture Conditions

Murine 3T3-A31 fibroblasts (Sigma-Aldrich, Darmstadt, Germany) were cultured in Dulbecco’s Modified Eagles’s Medium (Cat. No. D6429, Sigma-Aldrich, St. Louis, MI, USA) with 10% FBS (all purchased from Sigma-Aldrich, Darmstadt, Germany) and 1% penicillin/streptomycin (Sigma-Aldrich, Darmstadt, Germany). The cells were seeded on scaffolds at the density of 60,000 cells/scaffold and further cultured for 21 days. Samples were taken for analysis after 1, 7, 14, and 21 days of cultivation.

Data represent the mean ± SD of minimum 6 biologically independent experiments with technical triplicate each.

#### 2.7.1. Cell Viability, Cell Adhesion and Proliferation on 3D Scaffolds

The scaffolds used for in vitro assessment were made by freeze-drying method described in 2.1. The scaffolds were prepared in a 48 well plate and seeded with murine 3T3-A31 mouse fibroblasts as described above to evaluate their viability, adhesion, and proliferation. Staining of cell membranes with the fluorescent probe 3,3′-diethyloxacarbocyanine iodide DiOC3(6) (Invitrogen, Carlsbad, CA, USA) and cell nuclei with propidium iodide (PI, Sigma-Aldrich, Darmstadt, Germany) was used to detect the adhesion of cells on the scaffolds. Samples were fixed with frozen methyl alcohol (−20 °C) for 10 min and rinsed with PBS. Subsequently, DiOC3(6) (Cat. No. D273, Life Technologies, Eugene, OR, USA, 0.1–1 μg/mL in phosphate buffer saline (PBS), pH 7.4) was added and incubated with the samples for 45 min at room temperature. The samples were rinsed with PBS (pH 7.4), and propidium iodide (5 μg/mL in PBS, pH 7.4, Sigma-Aldrich, Saint Louis, CA, USA) was added for 10 min; they were then rinsed with PBS (pH 7.4) again and visualized using a confocal microscope (Zeiss LSM 5 DUO, Carl Zeiss Microscopy GmbH, Jena, Germany) on the 1st day. The areas of adhered cells were counted using Ellipse software (version 2.08, ViDiTo, Kosice, Slovak Republic).

To determine the ratio of living and dead cells 2′,7′-bis (2-carboxyethyl)-5(6)-carboxyfluorescein acetoxymethyl ester (BCECF-AM, Cat. No. B8806, Sigma-Aldrich, Saint Louis, MI, USA) was applied. The cells were visualized using a confocal microscope using λex = 488 nm and λem = 505–545 nm for BCECF or DiOC6(3) and λex = 560 nm and λem > 575nm for propidium iodide. On day 21, color-coded projections of cells in the depth of 200 µm were made to visualize penetration of cells into scaffolds from the surface (blue) to deep in the scaffold (red).

The extent of proliferation was evaluated from the samples embedded in a lysis buffer (0.2% *v*/*v* Triton X-100, 10 mM Tris (pH 7.0), and 1 mM EDTA) after three freeze-drying cycles using Quant-iT™ dsDNA Assay Reagent (Cat. No. Q33120, Invitrogen, Eugene, OR, USA). The dsDNA content was measured semi quantitatively using DNA standards included in the assay. Fluorescence was measured at λex = 485 nm and λem = 523 nm.

#### 2.7.2. Cell Metabolic Activity Analysis by the MTS Test

Cell metabolic activity was determined by CellTiter 96^®^ AQueous One Solution Cell Proliferation (MTS) assay (Promega, Madison, WI, USA). MTS is reduced to purple formazan by mitochondrial dehydrogenase in cells, indicating normal metabolism. 40 μL of MTS solution were added to the medium (200 μL), and the samples were further incubated at 37 °C for 2 h. 100 μL of the suspension were removed, and the optical density of the formazan was measured (λ of sample and reference was equal to 490 and 690 nm, respectively). The absorbance of samples incubated without cells was deducted from that of cell-seeded samples.

#### 2.7.3. Relative Collagen Type I mRNA Expression

All samples were cut into small pieces and RNA was isolated from them using RNeasy Mini Kit (74104, Qiagen, Hilden, Germany) following the manufacturer’s protocol. Nucleic acid concentration was measured using NanoQuant Plate (Tecan, Männedorf, Switzerland) and Infinite M200 Pro (Tecan, Männedorf, Switzerland). Subsequently, cDNA was synthesized using RevertAid First Strand cDNA Synthesis Kit (K1622, ThermoFisher Scientific, Vilnius, Lithuania). Polymerase chain reaction was performed using Light Cycler 480 (Roche, Basil, Switzerland). To each reaction, TaqMan™ Gene Expression Master Mix (4369016, Life Technologies, Vilnius, Lithuania), PCR Grade Water (03315959001, Roche, Mannheim, Germany), and depending on the desired gene TaqMan Gene Expression Assay containing both primer and probe were added. List of assays used: beta 2 microglobulin B2m (assay ID: Mm00437762_m1, 4331182), and collagen type I alpha 1 (assay ID: Mm00801666_g1, 4331182), all ThermoFisher Scientific, Pleasanton, CA, USA. The parameters of the reaction were set as follows: 1 cycle pre-incubation at 95 °C for 10 min, 45 cycles amplification each at 95 °C for 10 s, at 60 °C for 30 s and at 72 °C for 1 s, as a last step cooling for one cycle at 40 °C for 30 s. The data were analyzed using the relative 2-∆Cp method.

### 2.8. Angiogenic Properties by Ex Ovo CAM Assay

On the day of arrival (day 0), 36 fertilized chicken eggs were received from Henry Steward & Co. (UK), and were placed in a R-Com King Suro 20 digital egg incubator at 37.5 °C to allow the embryos to develop. On day 3, embryos were removed from their eggshells and transferred into 100 mL weighing boats with 2 mL PBS with 1% addition of penicillin streptomycin (PS). The weighing boats were then placed in Petri dishes containing 12 mL of distilled water and lids were placed over the weighing boats to conserve humidity. By removing the embryos from the shells, chick chorioallantoic membranes (CAMs) were exposed, allowing for clearer images to be obtained. The embryos were then transferred to trays in a humidified Binder Classic Line incubator and allowed to develop further at 37 °C. On day 7, a 3D scaffold sample, wet by 0.2 mL of PBS, was placed onto the surface of each CAM, and again allowed to develop in Binder incubator. Images were taken of the samples and surrounding vasculature on days 7 and 10–13, using Motorola USB microscope coupled with Microcapture imaging software. On day 13, the embryos were sacrificed, and the scaffold samples (including a small amount of the surrounding CAM) were explanted and fixed by placing in 3.7% formaldehyde for at least 24 h. To quantify angiogenic properties, the blood vessels growing perpendicularly (within ±45°) towards the scaffold were ranked, manually counted, and recorded. Analysis of vascular response was based on the total increase in blood vessels growing perpendicularly towards the sample, by subtracting the number of vessels seen on day 7 for a particular sample. All samples were blinded prior to counting to eliminate the risk of bias. The vasculogenic index corresponds to the number of newly created vessels between days 7 and 10as well as days 7 and 13 after fertilization.

### 2.9. In Vivo Evaluation in a Rabbit Model

**Figure 1:**
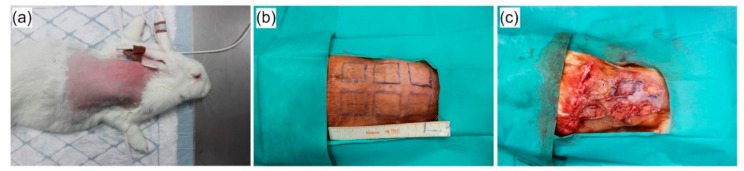
In vivo experiment on white New Zealand rabbits: (**a**) Animals were shaved at the places of wound surgery; (**b**) six defects were labeled on the shaved skin; (**c**) scaffolds (Coll, Coll/Chit and Coll/Chit_0.1 FGF2) were randomly implanted including empty defect-control group (**b**). Each group of implants was quadruplicated.

Four specific-pathogen free three-month old white New Zealand rabbits (*Oryctolagus cuniculus f. domesticus*) were supplied by a local production company approved by Ministry of Agriculture of the Czech Republic and housed at the Veterinary Research Institute (Brno, Czech Republic) in experimental stables certified by Ministry of Agriculture of the Czech Republic. The study was conducted according to the guidelines of the Declaration of Helsinki, and approved by the Institutional Review Board of Veterinary Research Institute (protocol code 12/2016 with approval from 21 April 2016) and by the Branch Commission for Animal Welfare of the Ministry of Agriculture of the Czech Republic (permission number 34715/2016-MZE-17214 from 15 June 2016). On arrival at the research facility, the rabbits were held for two weeks prior to the experimental procedure challenge and were individually housed in stainless-steel cages placed in isolated rooms with the controlled regime and independent ventilation. The rooms were kept at a temperature of 21 °C, relative humidity of 40 to 60%, and ventilation of approximately 15 air changes per hour. Prior surgery, the rabbits were anesthetized by intra-venous injection of Medetomidin at a dose of 0.5 mg/kg of body weight and Propofol at a dose of 8–15 mg/kg of body weight. Premedication and analgesia during the procedure was performed by subcutaneous injection of Butomidor at a dose of 0.1 mg/kg of body weight. Animals were shaved at the places of future wounds and antisepsis was performed by 2.5% solution of povidone iodine. Six full-thickness incisional wounds (including the panniculus carnosus) with dimensions of 3 × 3 cm were made symmetrically by scalpel excision on the depilated back of each rabbit. The split thickness skin grafts (STSGs) were made from the excised skin by removing the panniculus carnosus and the greater part of the reticular dermis with a scalpel blade. The scaffolds (Coll, Coll/Chit and Coll/Chit_0.1 FGF2) had thicknesses of 1.5 mm and diameters of 3 × 3 cm and were sterilized using ethylene oxide, grafted to the wound, and then covered by STSGs. The empty defect (without scaffold) was covered only by split thickness skin graft (STSG) and used as a control group (Figure 1). Each site treatment was provided in quadruplicate. Thereafter, the wounds were dressed with Vaseline gauze and sponge coverings, and an elastic bandage was then placed to secure the dressing.

The coverings were changed on day 5 after the initial application. Post-operative analgesia was used by subcutaneous injection of Meloxicam at a dose of 0.5 mg/kg of body weight once a day for a following three days. Bioptical samples were harvested at the 7th, 14th, and 21st day after grafting for histological evaluation. Relative quantification of mRNA expression of selected genes was provided at post-operative 21st day.

#### 2.9.1. Histological Assessment of Skin Formation

Histological staining of 5 mm thick paraffin sections was carried out by hematoxylin–eosin staining. We evaluated the presence of Coll/Chit biomaterial, inflammatory infiltrate, granulation tissue, newly formed fibrotic tissue, and multinucleated cells of foreign bodies on stained sections. Tissue sections were observed and photographed on an optical microscope (ZEISS Axio Scope.A1, Carl Zeiss Microscopy GmbH, Jena, Germany) at 7, 14 and 21 post-operative days.

#### 2.9.2. Gene Expression Analysis of Skin by RT-qPCR

To prevent potential destruction of RNA and changes in gene expression caused by other than experimental treatments, skin samples (each approximately 10 mg) cut into slices less than 0.5 cm thick were directly placed into a stabilization solution RNAlater (Qiagen, Hilden, Germany) after surgery, and stored at −70 °C until RNA isolation.

Prior to RNA isolation, skin tissues were homogenized in 1 mL of TRI Reagent RT (Molecular Research Center, Inc., Cincinnati, OH, USA) with 10 Zirconia/Silica beads of 2.3 mm in diameter (BioSpec Products, Inc., Bartlesville, OK, USA) using MagNA Lyser instrument (Roche Diagnostics, Mannheim, Germany). The RNA phase was obtained after separation using 4-bromoanisole treatment followed by 15 min centrifugation at 4 °C, and further applied in a purification procedure using RNeasy Kit (Qiagen, Tübingen, Germany) according to the manufacturer’s instructions in animal tissue protocol. In the final step, total RNA was obtained in 15 µL of RNase free water.

The RNA was reversely transcribed by M-MLV reverse transcriptase (200 U/µL, Invitrogen) and oligo(dT) RT primer (Generi Biotech, Hradec Kralove, Czech Republic) at 37 °C for 1.5 h. cDNA was stored at −20 °C until used. qPCR was performed in triplicate reactions on LightCycler 480 (Roche Applied Science, Penzberg, Germany)—one reaction contained 0.5 µL of cDNA (5× diluted), 1 µL of specific primer set in the amount of 10 pmol and 1.5 µL of master mix (QuantiTect SYBR Green PCR Kit, Qiagen, Tübingen, Germany). Primers for 9 chemokine and cytokine genes of interest (GOI) and 3 house-keeping genes as candidate reference genes (REF) (Generi Biotech, Hradec Kralove, Czech Republic, Table 2) were applied in the qPCR assay. References [47,48] show the origin of the primers used in this study and parameters for the primer design using NCBI software web tool Primer-BLAST (National Center for Biotechnology Information, Pike Bethesda, MD, USA) [49]. To detect potential contamination in each qPCR run, a non-template control was used. Using NormFinder software, out of the three candidate reference genes, hydroxymethylbilane synthase (HMBS) was selected as the reference gene with minimal gene expression variability among all samples tested. HMBS also served as a positive internal control in qPCR. The formula [1/(2^Cq GOI^)]/[1/(2^Cq REF^)] [50] was adapted in the qPCR assay to calculate the gene expression, including adjustment to the reference gene HMBS. The specificity of amplicons was confirmed using melting temperature test (LightCycler 480 software 1.5.1.62, Roche Life Science, Penzberg, Germany). The relative expression was calculated as a ratio of the gene expressions of the sample under the tested experimental treatment and the control sample (without the tested experimental material).

### 2.10. Statistical Analysis

For each parameter, mean values ± standard error of the mean (SEM, swelling ratio, degradations, mechanical testing, vasculogenic index and RT-qPCR) were calculated. *t*-test of two-samples assuming unequal variances (Excel, Microsoft Office 2021) was performed for mechanical testing. If not specified otherwise, biological data were evaluated using One-way ANOVA and the Student–Newman–Keuls method using SigmaStat 3.5 software (Systat Software, Inc., San Jose, CA, USA); *p*-values < 0.05 were considered to indicate statistically significant results.

## 3. Results

### 3.1. The Effect of Chitosan on Scaffold Swelling Ability

**Figure 2:**
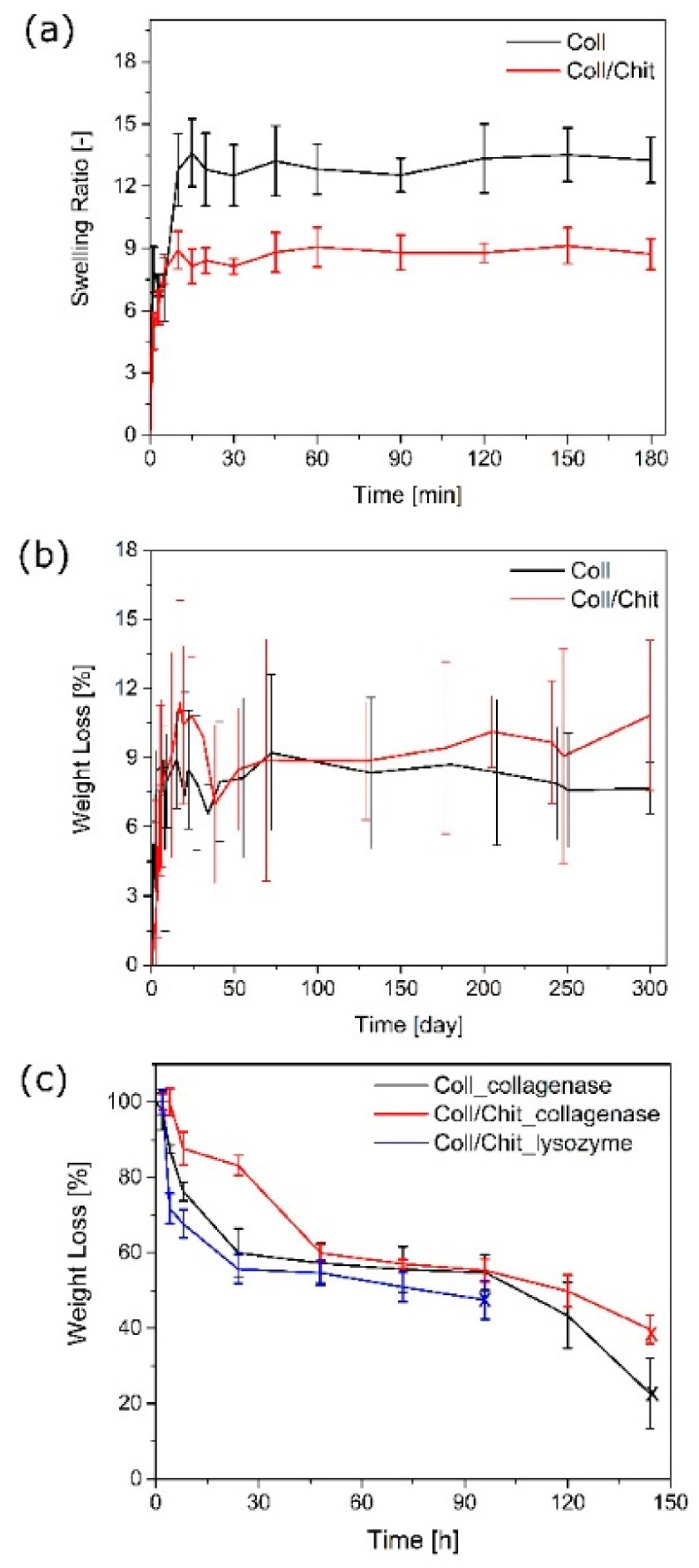
(**a**) Swelling behavior of Coll and Coll/Chit composite scaffold; (**b**) the dependence of the weight loss on the degradation time for Coll and Coll/Chit sponges; (**c**) mass loss of Coll and Coll/Chit scaffolds during the process of enzymatic degradation in PBS solution of either collagenase or lysozyme.

The ability of a scaffold to preserve water is an important aspect to evaluate its potential for skin tissue engineering. The swelling behavior of Coll scaffolds was obtained upon contact with distilled water. The rate of swelling decreased with time and reached a constant value, as illustrated in Figure 2a. Higher swelling ability was exhibited in the pure Coll scaffolds compared to Coll/Chit. Coll/Chit created a higher crosslinking network due to the mutual ionic and hydrogen bond interactions.

### 3.2. The Effect of Chitosan on Hydrolytic Degradation

The degradation of Coll scaffolds was simulated in an incubator in ultrapure water at 37 °C. Figure 2b shows the weight loss over time for Coll and Coll/Chit scaffolds, which were washed out with clean water during the degradation evaluation. The stability test confirmed the high efficacy of the crosslinking agent (EDC/NHS). The figure shows approximately 5 wt % of scaffold weight loss within first 3 days, while releasing unreacted Coll or Chit. This amount is low since the scaffold is thoroughly purified after the crosslinking procedure before the lyophilization step (see Section 2.1), thus providing a very high degree of crosslinking. Both Coll and Coll/Chit crosslinked scaffolds showed very high resistance to water, and no evidence of disintegration after a period of 300 days. Therefore, the enzymatic degradation of those scaffolds was shown to occur in a physiological environment.

### 3.3. The Effect of Chitosan on Enzymatic Degradation

Scaffolds based on Coll type I with or without Chit were evaluated during the enzymatic degradation treatment with collagenase. The graphical representation of weight loss of the scaffolds in time (up to 6 days) is shown in Figure 2c. As expected, the highest weight loss exhibited the pure Coll scaffold (77%). Even though the composite scaffold contained 50% of Coll, which is subject to collagenase degradation, its mass loss was beyond this content. The degradation of Coll/Chit scaffold was 60% of loss, which means that at least 10% of Chit was dissolved as well during this process.

### 3.4. The Effect of Chitosan on the Scaffold Stiffness

The biomechanical behavior of the Coll/Chit scaffolds was evaluated in the dry and hydrated state. A measurement, in which the sample is grasped at two ends and pulled, while the axial strain and stress are simultaneously measured was provided to determine the resistance of material to induced force. Coll and Coll/Chit scaffolds were liable to deformation test in both dry and wet conditions. In the dry measuring condition, the highest material resistance was attributed to the pure Coll scaffold, with a tensile strength of 87.8 ± 15.8 kPa, followed by the Coll/Chit, reaching only the half value of the Coll (45.9 ± 22.9 kPa). The addition of Chit contributed to a significant decrease in stress but resulted in more than twice higher elongation (3.1 ± 0.5 for Coll and 7.8 ± 0.2 for Coll/Chit). The tensile curves showed the similar trend as in the dry state, only a significantly smaller value of stress (8.2 ± 1.5 kPa for Coll/Chit) was required to cause permanent deformation of the scaffolds after 10 min of swelling. Pure Coll in hydrated environment has shown higher resistivity (27.1 ± 8.1 kPa) compared with Coll/Chit. There was also a significant increase in elongation of the material showing a similar trend as in the dry conditions, where Chit stretched the Coll fibers more in wet condition (9.4 ± 0.1% and 13.9 ± 0.1% for Coll and Coll/Chit, respectively).

**Figure 3:**
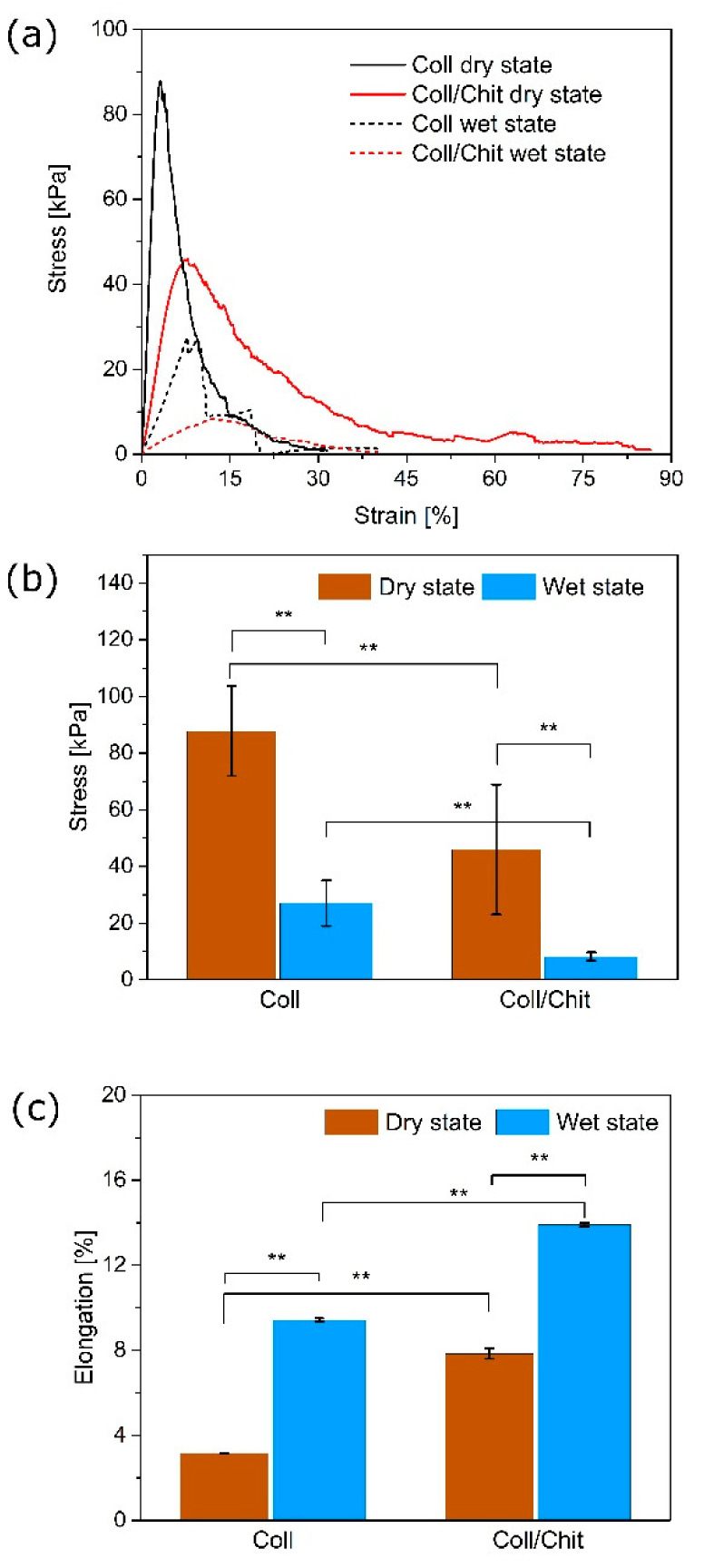
(**a**) Tensile curves of Coll and Coll/Chit scaffolds at dry and wet state; (**b**) the overview of stress values applied on Coll and Coll/Chit scaffolds at dry and wet state; (**c**) percentage elongation influenced by chitosan addition to collagen scaffold at dry and wet state; ** *p* < 0.01.

A graphical summary of the ultimate tensile strength points (stress values) and the elongation of scaffolds at different conditions are depicted in Figure 3b,c, respectively.

### 3.5. The Effect of FGF2-STAB^®^ on the Scaffold Structure

**Figure 4:**
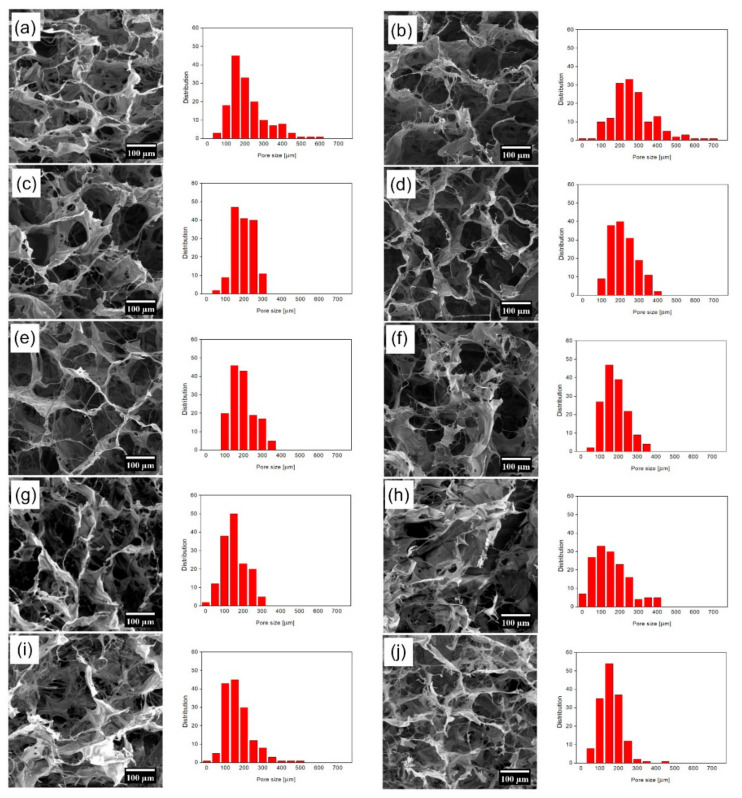
SEM visualization of prepared Coll/Chit scaffold enriched by increasing concentration of FGF2 (left—(**a**) Coll/Chit; (**b**) Coll/Chit_0.01 FGF2; (**c**) Coll/Chit_0.05 FGF2; (**d**) Coll/Chit_0.1 FGF2; (**e**) Coll/Chit_0.5 FGF2; (**f**) Coll/Chit_1 FGF2; (**g**) Coll/Chit_5 FGF2; (**h**) Coll/Chit_10 FGF2; (**i**) Coll/Chit_50 FGF2; (**j**) Coll/Chit_100 FGF2) and pore size distribution in the prepared scaffolds (right).

Three-dimensional (3D) porous scaffolds based on Coll and Chit with weight ratios of 1:1 enriched with increasing concentrations of human FGF2-STAB^®^ were successfully prepared. A homogenous network was formed by freeze drying, where an interconnected 3D porous structure within the samples was created. The morphologies of the obtained scaffolds are presented in Figure 4. As it is obvious in Table 1, the increasing concentration of FGF2-STAB^®^ in the Coll/Chit scaffolds led to the formation of more irregular pores with smaller size, where the smallest one exhibited the scaffold modified with 100 µg/mL of FGF2-STAB^®^ (100–220 µm).

With the FGF2-STAB^®^ concentration of 1 µg/mL, the Coll/Chit scaffolds started to include pores with size around 100 µm (and the distribution was close to Gaussian).

**Figure 5:**
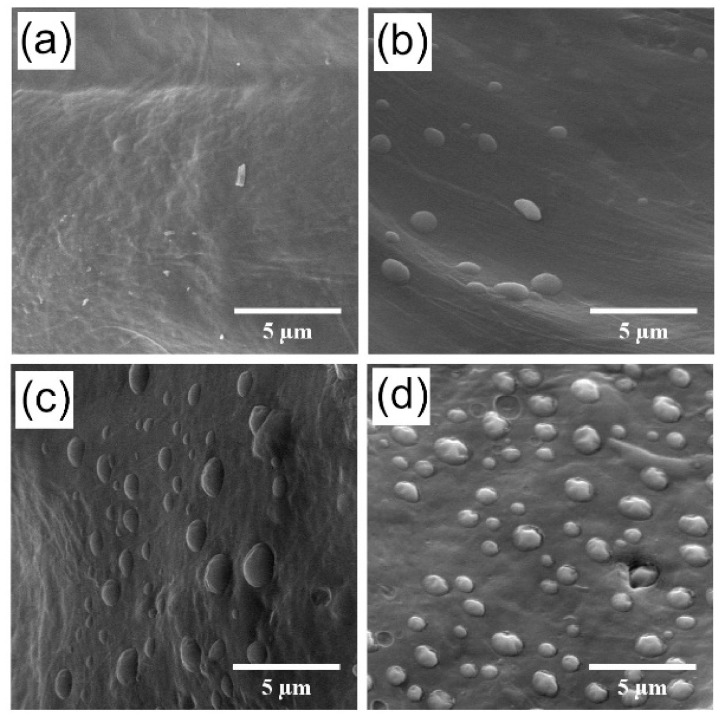
Representative SEM visualizations of prepared Coll/Chit scaffold enriched by increasing concentration of FGF2 with SEM magnification of 10k×: (**a**) pure Coll/Chit; (**b**) Coll/Chit_0.1 FGF2; (**c**) Coll/Chit_1 FGF2; (**d**) Coll/Chit_10 FGF2.

In addition, our results show that the addition of FGF2-STAB^®^ formed a spherical structure (size around 0.5–1 µm) on the smooth surface of Coll/Chit scaffolds, as can be seen in Figure 5.

### 3.6. The Effect of FGF2-STAB^®^ on Biological Properties

**Figure 6:**
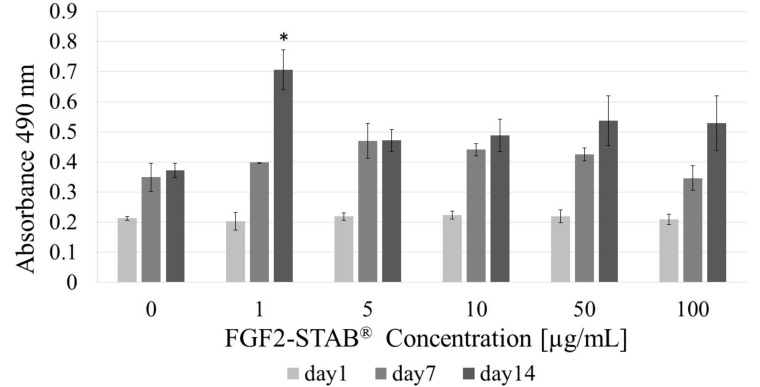
Fibroblast metabolic activity with increasing FGF2-STAB^®^ concentration (0, 1, 5, 10, 50, and 100 µg/mL) after 1, 7 and 14 days of incubation. Statistical significance (*p* < 0.05) marked with asterisk. On the 14th day the highest level was reached in the group with 1 µg/mL of FGF2-STAB.

To find the optimal FGF2-STAB^®^ concentration in Coll/Chit scaffolds, cytotoxicity studies were carried out on murine 3T3-A31 dermal fibroblasts, with different concentrations of FGF2-STAB^®^ (0; 1; 5; 10; 50 and 100 µg/mL) (Figure 6). The only significant result was observed on the day 14 in the group with 1 µg/mL FGF2-STAB where the cells reached the highest level of metabolic activity in comparison with other experimental groups.

**Figure 7:**
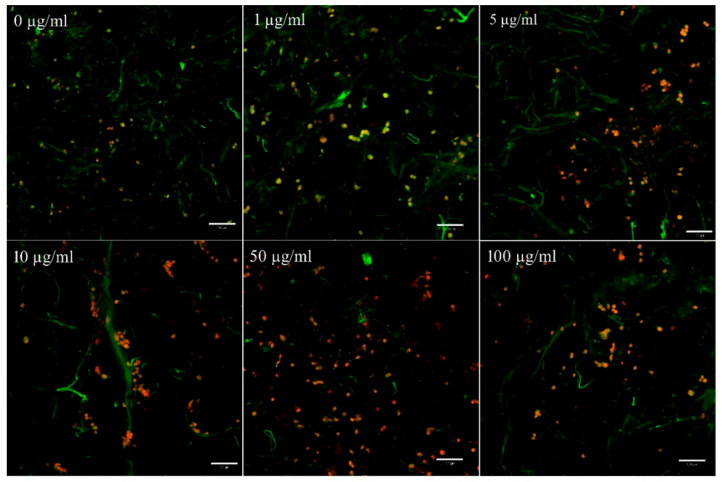
Confocal microscopy imaging of cell adhesion of 3T3 fibroblasts on Col/Chit scaffolds on day 1. Staining of cell membranes with the fluorescent probe 3,3′-diethyloxacarbocyanine iodide DiOC3(6) (green) and cell nuclei with propidium iodide (red) was used to detect the adhesion of cells on the scaffolds with different concentrations of FGF2-STAB^®^, a scale bar is 100 µm. Slight green autofluorescence of the Col/Chit scaffold is present.

Staining of cell membranes with the fluorescent probe 3,3′-diethyloxacarbocyanine iodide DiOC3(6) and cell nuclei with propidium iodide was used to detect the cell adhesion on the scaffolds (Figure 7).

**Figure 8:**
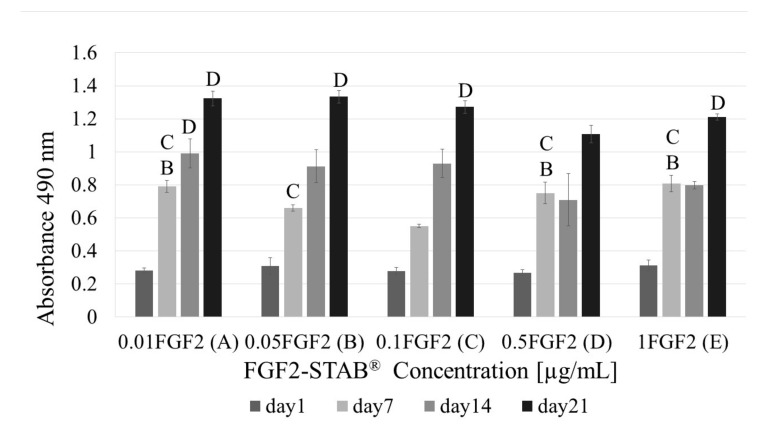
Fibroblast metabolic activity with increasing FGF2-STAB^®^ concentration (0.01, 0.05, 0.1, 0.5 and 1 µg/mL for **A**–**E**, respectively) after 1, 7, 14 and 21 days of incubation. Statistical significance for *p* < 0.05 is shown above columns.

As can be seen from Figure 6, cell metabolism is significantly higher at the concentration 1 µg/mL of FGF2-STAB^®^ especially at day 14 showing enhanced cell proliferation. Therefore, the higher concentration of growth factor does not have to be used for further experiments. The effective low concentration of FGF2-STAB^®^ might be dependent on the hyper stable FGF2 structure enabling full protein activity up to 20 days at 37 °C. Therefore, for further concentration optimization, cytotoxicity was evaluated for FGF2-STAB^®^ concentrations 0.01; 0.05; 0.1; 0.5 and 1 µg/mL. Figure 8 shows the metabolic activity measured via MTS assay. As can be seen, the best metabolic activity reveals Coll/Chit scaffolds enriched up to 0.1 µg/mL of FGF2-STAB^®^.

**Figure 9:**
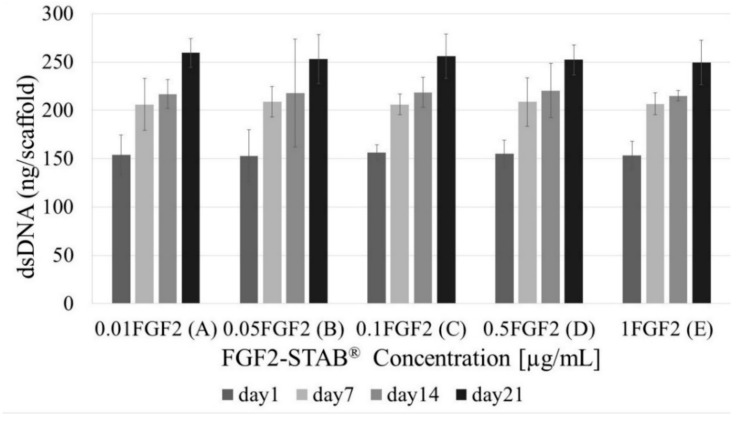
dsDNA assay with increasing FGF2-STAB^®^ concentration (0.01, 0.05, 0.1, 0.5 and 1 µg/mL, for **A**–**E**, respectively) after 1, 7, 14 and 21 days of incubation. No statistical significance was observed.

As for the dsDNA assay, no statistical difference was found within all tested concentrations as is shown in Figure 9 proving that all samples had similar cell numbers over time.

**Figure 10:**
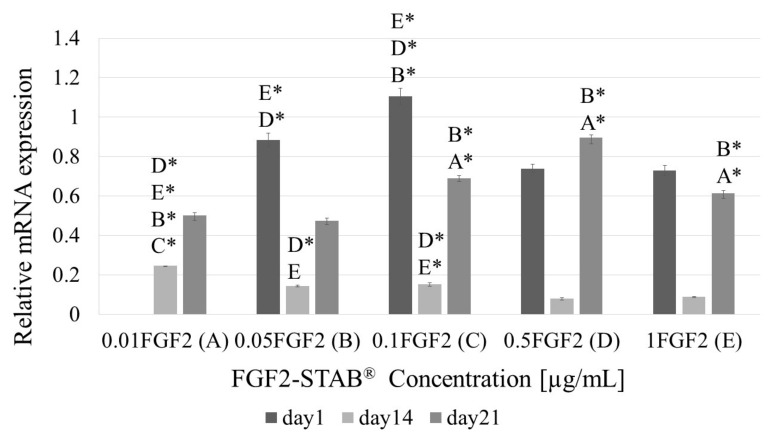
Relative collagen I mRNA expression of samples with increasing FGF2-STAB^®^ concentration (0.01, 0.05, 0.1, 0.5 and 1 µg/mL, for **A**–**E**, respectively) after 1, 7, 14 and 21 days of incubation. Statistical significance for *p* < 0.05 or * *p* < 0.001.

However, there was a statistical significance in the Coll I expression by 3T3-A31 fibroblasts as shown in Figure 10. While the concentration 0.1 µg/mL of FGF2-STAB^®^ in Coll/Chit samples exhibited the best Coll expression within the first 24 h, in long-term study (21 days) the best sample seem to be scaffold involving 0.5 µg/mL of FGF2-STAB^®^.

**Figure 11:**
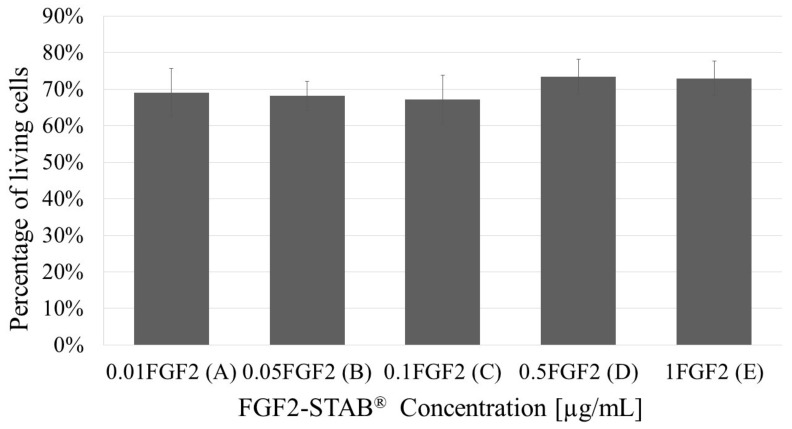
The live/dead assay in day 21 of samples with increasing FGF2-STAB^®^ concentration (0.01, 0.05, 0.1, 0.5 and 1 µg/mL, for **A**–**E**, respectively). No statistical significance.

The viability of cells at day 21 taken from the live/dead assay has not shown any statistical difference among the samples (Figure 11).

**Figure 12:**
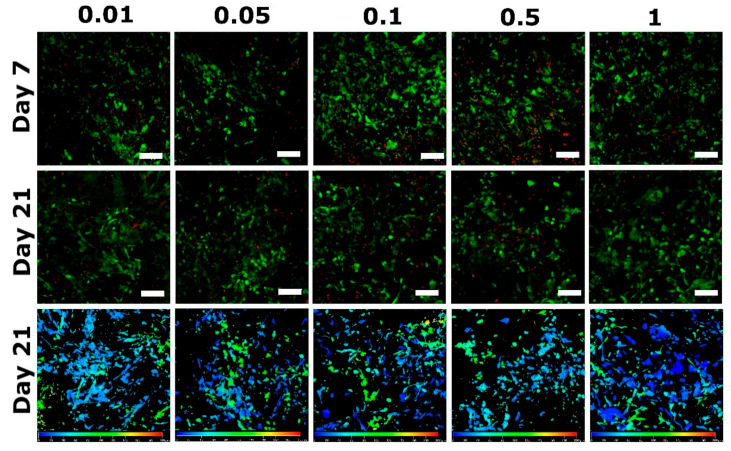
The live (green)/dead (red) cell distribution on the day 7 (first row) and day 21 (second row) including deep color-coded projection from confocal microscopy (bottom row) from the surface (blue) to the depth of 200 µm (red) on day 21. Cells were seeded on samples with increasing FGF2-STAB^®^ concentration (0.01, 0.05, 0.1, 0.5 and 1 µg/mL), a scale bar is 100 µm.

The viability of cells after 3 weeks of incubation with Coll/Chit samples is about 70% in all measured concentrations of FGF2-STAB^®^. The live (green) and dead (red) cells distribution within the samples in day 7 and 21 shows Figure 12. The deep projection from confocal microscope in 200 µm depth shows overall cells distribution within the whole sample volume.

Based on the cytotoxicity and biocompatibility evaluation, for further ex ovo and in vivo testing, only Coll/Chit scaffolds involving 0.1 µg/mL of FGF2-STAB^®^ were used.

### 3.7. The Effect of FGF2-STAB^®^ on Angiogenesis Evaluation

**Figure 13:**
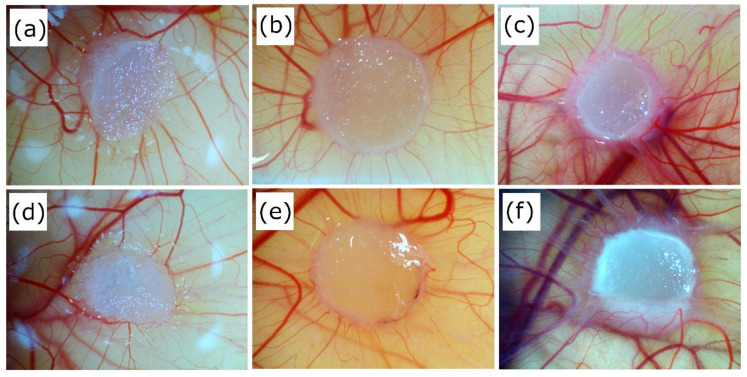
Coll/Chit (**a**–**c**) and Coll/Chit_0.1FGF2 (**d**–**f**) samples implanted on CAM at day 7 (**a**,**d**), 10 (**b**,**e**) and 13 (**c**,**f**). No significant differences were found.

**Figure 14:**
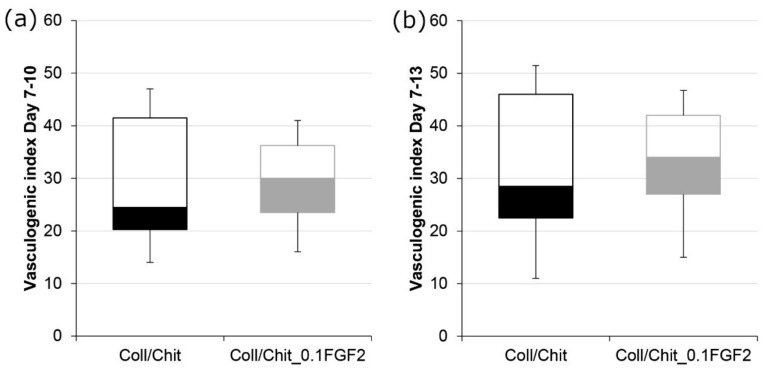
Box plot of vasculogenic index: (**a**) between day 7 and day 10 for Coll/Chit samples without FGF2-STAB^®^ and with 0.1 µg/mL of FGF2-STAB^®^; (**b**) between day 7 and day 13 for Coll/Chit samples without FGF2-STAB^®^ and with 0.1 µg/mL of FGF2-STAB^®^.

Using CAM assay evaluation, we can measure functional blood vessel formation ex ovo. Figure 13 depicts the increase in the number of vessels growing perpendicularly towards each sample. As it can be seen in Figure 14, the Coll/Chit scaffolds enriched by FGF2-STAB^®^ revealed a slightly higher vasculogenic index, 29.3 ± 8.3 and 38.0 ± 10.6 between day 7–10 and day 7–13, respectively, compared to the scaffolds without FGF2-STAB^®^ (28.7 ± 12.4 and 33.8 ± 15.8, respectively). Since pure Coll/Chit scaffolds are able to highly promote vascularization in this model, the addition of FGF2-STAB^®^ was not able to show a significant effect on top of the angiogenic capacity of the Coll/Chit scaffolds.

### 3.8. The Effect of FGF2-STAB^®^ on In Vivo Biocompatibility

**Figure 15:**
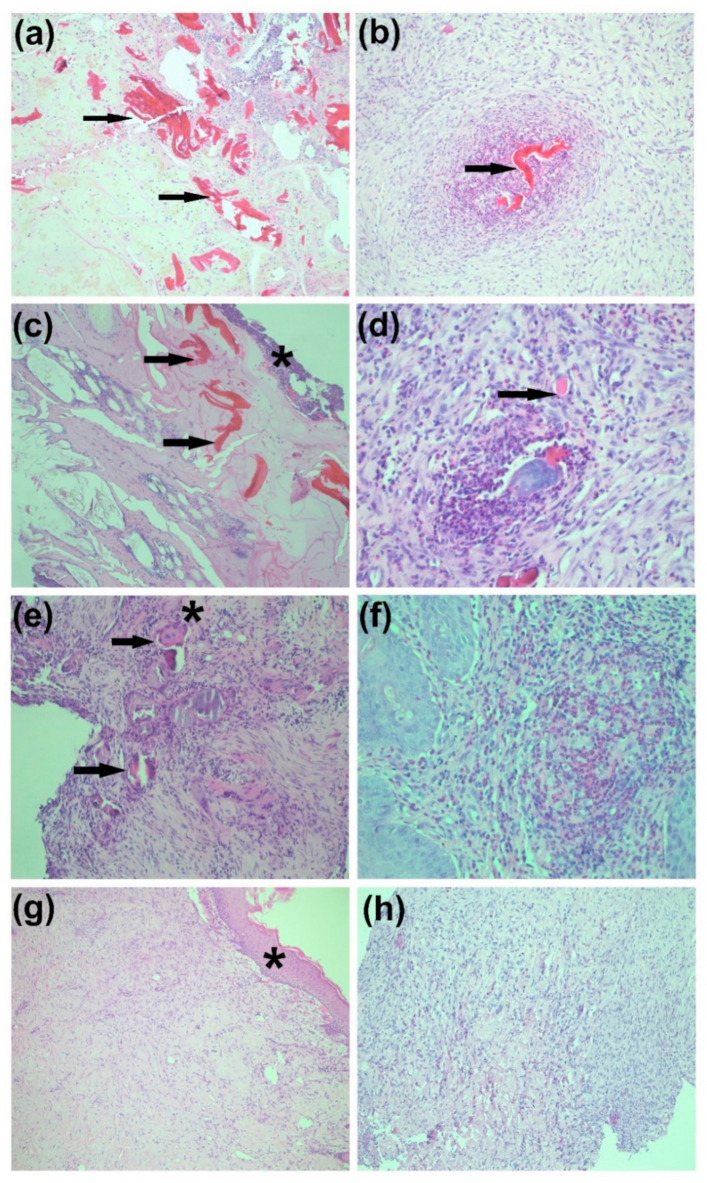
Histological overview of a hematoxylin-eosin-stained section of rabbit skin after implantation of Coll/Chit without FGF2 after 7 days (**a**); 14 days (**c**) and 21 days (**e**); and after implantation of pure dermo–epidermal graft after 21 days (**h**). (**a**) Some Coll/Chit biomaterial residue with mixed inflammatory cell infiltrate in the deeper tissue layers. No significant evidence of resorptive reaction. (**c**) Evidence of Coll/Chit scaffold residues with mixed inflammatory infiltration in the environment. Epithelial layer is present on the surface. (**e**) Presence of Coll/Chit biomaterial residue at the defect site, with mixed inflammatory cell infiltrate and granulation tissue. Numerous multinucleated foreign body giant cells are visible ✱. (**h**) Newly formed tissue at the site of a previous defect without significant inflammatory infiltration. Magnification is 100× for all images. Histological overview of a hematoxylin-eosin-stained section of rabbit skin after implantation of Coll/Chit_0.1FGF2 followed by time intervals of 7 days (**b**), 14 days (**d**), and 21 days (**f**,**g**). (**b**) Coll/Chit scaffold residues with mixed inflammatory infiltration in the environment, magnification 100×. (**d**) The Coll/Chit biomaterial residue with mixed inflammatory infiltration in the area and with the formation of granulation tissue and one type of huge multinucleated cell of foreign bodies (modified macrophages—these cells are commonly found at the site of biomaterials and indicate of granulation tissue formation), which is a sign of very good chitosan resorption ability, magnification 100×. (**f**) Implant site after complete chitosan resorption with mixed inflammatory infiltration and formation of granulation tissue in the area, magnification is 100×. (**g**) Newly formed tissue at the site of a previous defect without significant inflammation and infiltration, superficial epithelial layer ✱, magnification is 200×.

**Figure 16:**
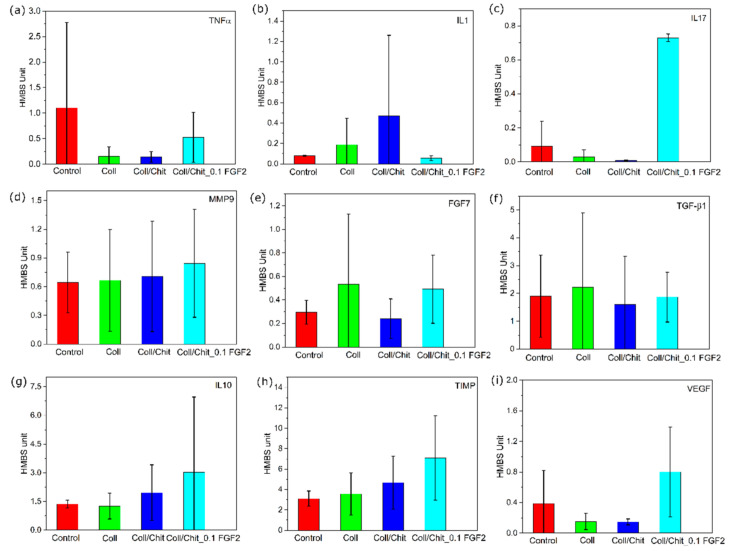
Expression of mRNA of cytokines: (**a**) TNFα; (**b**) IL1; (**c**) IL17; (**d**) MMP9; healing proteins (**e**) FGF7 and (**f**) TGF- β1; (**g**) anti-inflammatory cytokine interleukin 10 (IL10); (**h**) tissue inhibitors of metalloproteinases (TIMP-1); (**i**) vascular endothelial growth factor (VEGF) of sites treated with scaffolds compared to non-treated control site at post-operative day 21. Results are expressed as relative mean ± SD of HMBS (house-keeping gene) units. No statistical significance.

In the next phase of testing the Coll/Chit based scaffolds, four New Zealand white Rabbits (*Oryctolagus cuniculus f. domesticus*) were used as an animal model for full skin thickness replacement. Consistent with in vitro studies, in vivo results demonstrated high biocompatibility of implanted either pure Coll, Coll/Chit, or Coll/Chit_0.1FGF2 matrices with no evidence of defect healing disorders 21 days after implantation. In Figure 15a–e histological images show the presence of nonresorbed Coll/Chit remnants. The control group defect healed only by the application of STSG is shown in Figure 15h. The defect is replaced by fibrous tissue of neodermis. No hypertrophic scarring or long-term foreign body reaction induced by the implanted material was demonstrated, as shown in histological images in Figure 16b,d,f,g with Coll/Chit_0.1FGF2.

### 3.9. The Effect of FGF2-STAB^®^ on Gene Expression by RT-qPCR

Figure 16 shows the expression of mRNA of pro-inflammatory (TNFα, IL1β, IL17 and MMP9) and anti-inflammatory (IL10 and TIMP1) cytokines were not affected by any material used when compared to untreated control at post-operative day 21. Although a limited number of samples was analyzed for the expression of mRNA of healing-associated proteins (VEGF, TGF-β1, FGF7),

Expression of mRNA for anti-inflammatory cytokine interleukin 10 (IL10), tissue inhibitors of metalloproteinases (TIMP-1) and vascular endothelial growth factor (VEGF) are depicted in Figure 16g–i, respectively. Results expressed relative to the house-keeping gene (HMBS) show slightly higher expression of IL10, TIMP1 and VEGF genes in sites treated with Coll/Chit scaffolds enriched with FGF2-STAB^®^ protein. Comparing to pure Coll or Coll/Chit scaffold the highest increase shows value of mRNA for VEGF supporting vascularization for scaffold modified with FGF2-STAB^®^ protein.

## 4. Discussion

Composite scaffolds have shown several advantages compared to one-component scaffolds, such as the ability to tailor their mechanical properties, physico-chemical properties, ultrastructure and surface topography, growth factors or bioactive drug binding capacity, degradation, drug release, cell adhesion, proliferation, and differentiation [51,52,53,54]. Moreover, modification of scaffolds with growth factors significantly affects cell adhesion, migration, proliferation, differentiation, and ECM synthesis. Drug delivery systems based on degradable polymers and growth factors allow longer delivery of growth factors, however, the applied dose is usually high and growth factors have to be applied daily due to fast degradation of the protein [55,56]. The stability of natural growth factors is often limited to several hours. Human or zebrafish FGF2 lose their activity after a 24 h incubation at 37 °C. The instability was caused mainly by aggregation of the FGF2, which could be decreased by the addition of heparin or heparan sulphate [57]. Therefore, the stabilization of growth factors is desired for their long-term effect along with their lower dosage and lower side effects. Different attempts to stabilize FGF2 have been studied. They include conjugation to heparin or heparin-like molecules, coacervation, chemical modifications, genetic engineering, physical barrier strategies, entrapment in hydrogels, microencapsulation and adsorption [58].

FGF2 plays a role in embryonic development, tissue homeostasis, wound healing, and cancer. The role of FGF2 in the therapy of lung, neural, or vascular diseases, wound healing and diabetes or myocardial revascularization has been already studied [59,60,61].

FGF2-STAB^®^ is a human recombinant protein that is stable at 37 °C degrees for 20 days [45,62]. The increased stability of FGF2 decreases the dependence of FGF2 on heparin [62] allowing lower doses to be used in a scaffold and the retention of dose dependent FGF2 biological activity for few weeks in in vitro studies as well as in vivo. It has a positive effect on pluripotency of embryonic stem cell at cells up to 100 ng/mL while lower levels of 1–10 ng/mL are effective for other cell types [63]. Kanematsu et al. [64] revealed that FGF2 spontaneously interacts with type I Coll solution and sponges under in vitro and in vivo physiological conditions. FGF2 complexed with Coll by electrostatic ionic interactions showed resistance to trypsin digestion and is, therefore, protected from the proteolytic environment by the Coll. FGF2 incorporated in a Coll sponge sheet was sustainably released in the mouse subcutis according to the biodegradation of the sponge matrix and exhibited local angiogenic activity in a dose-dependent manner. Coll produced from myofibroblasts may function as a transient reservoir of FGF2 until the remodeling process accompanied by neovascularization of the wound scar is accomplished. The manner of controlled FGF2 release from Coll matrices was observed in several studies. It was found that growth factors can bind to the Coll matrix and then gradually release them during its degradation. The rate of release can be influenced by a number of factors, not only by the preparation of Coll matrices (crosslinking, lyophilization), but also by the modifications using different types of additives (e.g., heparin, gelatin, Chit, cellulose) [65,66]. For example, Wu et al. [66] determined the FGF2 release profile from the FGF2-Coll matrices using an ELISA assay. Preabsorbed FGF2 released from the non-crosslinked Coll group with an initial burst phase of 36.9% during the first 24 h followed by FGF2 rapid release ended by day 14 with 100% when the Coll matrix had degraded into small debris. Contrarily, the EDC/NHS crosslinked Coll exhibited relatively small initial burst releases of 19.5% FGF2 sustained controlled release over 37 days.

Since the collagenous material is degraded in vivo by complex reactions of proteolytic enzymes, the in vivo release profile of FGF2 from the sponges could not be predicted only by experiments simulating physiological environment. As we have proved, the integrity of EDC/NHS crosslinked Coll/Chit scaffold 3D network under hydrolytic conditions remained very stable (longer than 300 days at 37 °C), contrary to the enzymatic degradation study, where the scaffold was disturbed as the Coll degraded using collagenase within 140 h and smaller pieces of Chit were separated from the scaffold. This was confirmed by the visible small chunks presented in the solutions with composite scaffolds. No chunks were observed in the solution when pure Coll was tested. However, where lysozyme was applied instead of collagenase, approximately 50% of Chit degraded from the composite scaffold. Higher Coll/Chit stability and the lower water-binding ability in comparison to the Coll scaffold could be attributed to both the hydrophilicity and the maintenance of their 3D structure. As the Coll is crosslinked with Chit it decreased the swelling ratio, accompanied with reduced amount of free bind hydrophilic groups.

Therefore, the FGF2 release from Coll/Chit scaffolds was examined as the FGF2 biological activity both in vitro on murine 3T3-A31 mouse fibroblast cells and in vivo on New Zealand white rabbits. Metabolic activity was tested on a broad range of FGF2-STAB^®^ concentrations from 0 to 100 µg/mL. The FGF2-STAB^®^ concentration of 1 µg/mL showed a significant increase in metabolic activity on day 14. Subsequently, the testing of lower concentrations showed improved cell metabolic activity at 0.01and 0.05 µg/mL compared to higher concentration of FGF2-STAB^®^, which is likely caused by the high stability of FGF2-STAB^®^. Interestingly, cell proliferation was not affected by lower concentrations of FGF2-STAB^®^. In 3D scaffolds, the cell proliferation and differentiation are also affected by nutrition supply, which is influenced by diffusion rate, and the physical-chemical properties of the scaffolds. Coll I expression was higher in samples with 0.1 and 0.5 µg/mL of FGF2-STAB^®^. This is contrary to previous studies which have shown FGF2 decreased Coll I expression and decreased scar formation [60]. Thus, the lower dosage of FGF2-STAB^®^, which has a positive effect on metabolic activity of fibroblasts but with no effect on Coll synthesis, may be more beneficial for wound healing. This is in good agreement with [63].

The mechanical properties of the prepared scaffolds are critical for handling during surgery [67]. Therefore, the addition of Chit into the scaffolds was chosen to improve the overall mechanical properties of the scaffolds, which is in good agreement with published results [68,69]. Chit of higher molecular weight significantly improved biomechanical properties of composite Coll/Chit scaffold compared to Chit of low molecular weight [70]. Moreover, Chit can greatly support the material stretching long before the start of nonreversible deformation [71,72]. As shown in Figure 3a, every tensile curve is characterized by three different regions. The first linear region represents the continually rising stress applied to the material accompanied with rising material tension. Its stiffness increases rapidly as the Coll and Chit fibers are straightened and begin to carry a major part of the load. In this moment, the material acts as elastic. The linear region ends when the material no longer puts resistance to the applied stress, called ultimate tensile strength. At this point, the straightness of Coll and Chit fibers is maximized, the amount of stress applied on the material is too high and ultimately shows fiber failure. In the last region, the fibers are slowly disconnected by gradual tearing. Overall, the Chit addition as well as hydrated conditions created a region of plastic deformation, as can be seen in Figure 3a. The Coll only scaffold in a dry state showed that ultimate tensile strength can be equal to the yield point, which indicates the limit of elastic behavior. After this point, no plastic deformation occurred, but instead a tearing of the material was observed.

SEM revealed that the addition of FGF2-STAB^®^ visually changed the microstructure of the prepared Coll/Chit scaffolds. The scaffold microstructure is mostly formed by interconnecting pores. Pure Coll/Chit scaffolds exhibited a honeycomb-like structure with the pore size in the range of 140–340 µm that is in a good agreement with the results published by Martínez et al. [73] as well as Rezaii et al. [74]. We assume that higher addition of FGF2-STAB^®^ resulted in the filling pores in the scaffold by FGF2-STAB^®^ complexation leading to the decrease of scaffold pore size.

Concerning the full-skin thickness regeneration, the formation of new blood vessels from the existing vasculature plays a fundamental role. Since Coll/Chit_FGF2-STAB^®^ scaffolds contain various bioactive agents, which have the potential to modulate angiogenesis, a CAM assay was used to test the angiogenic potential in an ex ovo environment. In tissue engineering research, angiogenesis is essential to promote microvascular networks inside engineered tissue constructs, which maintain adequate tissue oxygenation, nutrient transfer, and waste removal. According to our previous study [23], Coll and Chit showed a positive synergistic effect performing in vitro experiments on scaffolds with fibroblasts. As it was expected, the pure Coll/Chit scaffolds supported angiogenesis ex ovo, therefore we were unable to observe any significant difference between the scaffolds with and without FGF2-STAB^®^ types as both displayed positive effects. Here, we used purified and stabilized FGF2-STAB^®^, which is considered as a growth factor affecting the expression of vascular endothelial growth factor and other pro-angiogenic cytokines [75]. Our finding that FGF2 stimulates angiogenesis follows other studies discussing the positive effects of FGF2 on angiogenesis, and stimulation of VEGF expression [76,77,78]. In in vivo models of guinea pig, Coll type I sponges covered with FGF2 or seeded with fibroblasts showed increased tensile strength and increased reepithelization of the wound 15 days after excision. Coll type I sponge-treated group showed increased tensile strength of the wound [79]. Interestingly, PDGF-loaded Coll/Chit scaffold led to significantly higher Coll type I amount in newly formed tissue compared to PDGF-loaded Chit scaffold on day 4. Both PDGF-treated groups showed significantly increased Coll synthesis compared to untreated defect [80]. Similarly, we have observed higher mRNA Coll I expression in Coll/Chit_0.1 FGF2 compared to Coll/Chit_0.5 FGF2 and Coll/Chit_1 FGF2 samples on day 1 and 14. In addition, complete epithelialization was found in PDGF-loaded Coll/Chit scaffolds with no signs of inflammation, while PDGF-loaded Chit scaffold showed stratified squamous epithelial layer formation and loose connective stroma tissue with capillaries on day 10. This is in accordance with our results of Coll/Chit_0.1 FGF2 group which does not contain significant inflammation and contained formed epithelium [80].

Tissue damage can lead to inflammation by the release of so-called damage-associated molecular patterns (DAMPs). This is associated with injuries of the skin by various external impacts including mechanical damage, and physical or chemical effects. DAMPs are recognized by membrane receptors, toll-like receptors or receptor RAGE for advanced glycation end products. These interactions lead to cascade of signal transmission and nuclear translocation of transcription factors such as NF-κB. This activation leads to transcription of mRNA for many pro-inflammatory cytokines, for example IL1β, IL17, and matrix metalloproteinases (MMPs). The mRNA expression for these genes was influenced very little when defects were treated with any of combination. Another pro-inflammatory protein produced after nuclear translocation of NF-κB is TNFα. It is produced predominantly by activated macrophages and is involved in the up regulation of inflammatory reactions. Its mRNA expression seems to be down-regulated by all combinations of materials used for treatment of the defects.

For healing process, it is crucial that inflammation is strictly controlled. Down-regulation of the inflammatory reactions is mediated in several ways—from the production of glucocorticoids by the adrenal cortex to a production of anti-inflammatory cytokines including interleukin-10. IL10 is one of the most potent agents able to re-polarize inflammatory M1 macrophages to anti-inflammatory M2 macrophages with healing and building properties [81,82]. Another important anti-inflammatory action is down-regulation of the activity of MMPs is via binding to a family of homologous proteins referred to as the tissue inhibitors of metalloproteinase TIMP [83]. One of the members of TIMP family—TIMP-1 was shown to also have cell growth promoting activity on human keratinocytes and several other cell types [84,85]. Expression of mRNA for both of these proteins was up-regulated when Coll/Chit material was supplemented with FGF2-STAB^®^ protein. Another important part of the healing process is also associated with the production of a family of growth factors—fibroblast growth factors (FGF), transforming growth factors (TGF) and vascular endothelial growth factor (VEGF)—all being measured in our study. FGF7 and TGF-β1 mRNA expression was not influenced by FGF2-STAB^®^ supplementation. In the case of VEGF, FGF2- STAB^®^ supplementation led to an increase tendency. VEGF is produced by a cell suffering from lack of oxygen via a hypoxia-inducible factor [86]. However, macrophages and particularly M2 polarized macrophages can contribute to enhanced neovascularization by production of VEGF [87]. The question is whether FGF2-STAB^®^ can polarize macrophages to M2 mode, as it was shown in the case of FGF9 [88]. Very recently, it was shown that also FGF2 could be a major modulator in macrophage polarization, at least in tumor development [89]. Change of expression of IL10, VEGF and TIMP by Coll/Chit supplemented with FGF2-STAB^®^ is in correlation with the fact, that 21 days after surgery Coll/Chit is not present in histological sample and anti-inflammatory and healing associated processes prevailed. On the other hand, by Coll/Chit with no FGF2-STAB^®^, healing—associated processes were delayed as Coll/Chit remnants still could be detected by histological analyses. All samples revealed very good Chit resorption and formation of granulation tissue in the defect area was observed. Regarding wound closure, no visible differences were found among the tested scaffolds, which may be due to the very fast skin regeneration of rabbit animal model. Therefore, for subsequent studies, an animal model of a Large White pig was used, where various biopolymers and the efficacy of FGF2-STAB^®^ in the concentration range from 0.1–10 µg/mL for the healing and neovascularization of full-thickness skin defects were evaluated. The results of the mentioned study will be published soon.

## 5. Conclusions

In the proposed work, collagen-based scaffolds modified with antibacterial Chit fibers and hyper-stable FGF2-STAB^®^ protein were evaluated via in vitro, ex ovo and in vivo tests. The main goal was to prove the safety of the applied stabilized FGF2-STAB^®^ protein as well as to demonstrate its efficacy in healing and neovascularization of full thickness skin regeneration in a rabbit animal model. Regarding cell adherence, viability, and cell proliferation, in vitro tests prove non-cytotoxicity and good biocompatibility of the scaffolds used. Moreover, good neovascularization evaluated within the CAM assay was confirmed on Coll/Chit scaffolds either with or without FGF2-STAB^®^. Finally, an in vivo study on rabbit animal model revealed superiority of scaffold with FGF2-STAB^®^ in terms of scaffold bioresorption, minimal inflammation, and excellent granulation on skin tissue reconstruction. Gene expression for mRNA of different pro-inflammatory (IL1β, IL17, MMP9, and TNFα), anti-inflammatory (IL10, TIMP1), and pro-healing proteins (TGFβ, FGF7, and VEGF) proved the safety and positive effect of Coll/Chit scaffolds with FGF2-STAB^®^ on skin regeneration, especially its dermal part. Our study provides the necessary base for further evaluation of the scaffold for biomedical and clinical applications in the fields of tissue engineering and regenerative medicine.

## Figures and Tables

**Table 1:** Sample labeling, the used FGF2 concentration and pore size of prepared scaffolds.

Sample Labeling	C_FGF2_ [µg/mL]	Pore Size [µm]
Coll/Chit	0	240 ± 100
Coll/Chit_0.01 FGF2	0.01	250 ± 100
Coll/Chit_0.05 FGF2	0.05	240 ± 70
Coll/Chit_0.1 FGF2	0.1	220 ± 60
Coll/Chit_0.5 FGF2	0.5	220 ± 60
Coll/Chit_1 FGF2	1	210 ± 60
Coll/Chit_5 FGF2	5	180 ± 60
Coll/Chit_10 FGF2	10	170 ± 90
Coll/Chit_50 FGF2	50	170 ± 70
Coll/Chit_100 FGF2	100	160 ± 60

**Table 2:** Gene specific primer sets used in qPCR mRNA detection.

Gene	Forward Primer Reverse Primer	Function
TNF-α	CTCTGCCTCAGCCTCTTCTCTT	tumor-necrosis factor—alpha
	AGGTTGTTTGGGGACTGCTCT	pro-inflammatory
IL-1ß	ACAACAAGTGGTGTTCTCCATGA	Interleukin—1 beta
	TTTCATCACGCAGACAGGTACA	pro-inflammatory
IL-17	ACCACATGAACTCTGTCCCAATC	Interleukin—17
	CCTACAGCCACCAGCATCTTC	pro-inflammatory
MMP-9	CACTGGGCTTGGATCACTCCTC	matrix metalloproteinasis—9
	GGGTTAGGACCATATAGATGCTGGA	pro-inflammatory
IL-10	TTCTGTGCCTGACCACACTTTC	Interleukin—10
	CTAGGAGTCTCTGGAACACTCGG	anti-inflammatory
TIMP-1	GTTTCTCATCGCTGGACAACTGC	tissue inhibitor of metalloproteinasis—1
	ACGAAACTGCAAGTCGTGATGTG	anti-inflammatory
TGF-ß1	TTCCCCTCCGAAAATGCCATCC	transforming growth factor—beta 1
	CACTCTGGCTTTTGGGTTCTGC	wound healing
VEGF-C	GCTTCTTGTCTCTGGCGTGTTC	vascular endothelial growth factor—C
	CCTACATAAGCCTTGGCCTCCTC	wound healing
FGF-7	ATCCTGCCAACTTTGCTCTACAGA	fibroblast growth factor—7
	CTGGAGTCATGTCATTGCAAGCT	wound healing
HPRT-1	TGAAACTGGAAAAGCAAATACAAAG	hypoxanthine phosphoribosyltransferase—1
	CGATGTCAATGAGACTCCTGATG	house-keeping gene
GAPDH	GAATCCACTGGCGTCTTCAC	glyceraldehyde-3-phosphate dehydrogenase
	CGTTGCTGACAATCTTGAGAGA	house-keeping gene
HMBS	CAGCCATGAAGGATGGGCAGCTGTAC	hydroxymethylbilane synthase
	TGCTGGCCTGCATGGTCTCTTGC	house-keeping gene

Orientation of primers from 5′ to 3′.

## Data Availability

The data presented in this study are available on request from the corresponding author.
